# A simple plant high-molecular-weight DNA extraction method suitable for single-molecule technologies

**DOI:** 10.1186/s13007-020-00579-4

**Published:** 2020-03-14

**Authors:** Zhigang Li, Stephen Parris, Christopher A. Saski

**Affiliations:** Department of Plant and Environmental Sciences, College of Agriculture, Forestry and Life Sciences, Clemson University, Clemson, SC 29634 USA

## Abstract

**Background:**

High-molecular-weight and pure DNA is crucial for high-quality results from 3rd generation DNA Analyzers and optical mapping technologies. Conventional nuclei isolation methods for preparing high-molecular-weight genomic DNA from plant tissues include the preparation of protoplasts or embedding nuclei in an agarose matrix with subsequent manipulations via electro-elution or pulsed-field gel electrophoresis.

**Results:**

In this method, plant nuclei are isolated by physically grinding tissues and reconstituting the intact nuclei in a unique Nuclear Isolation Buffer (NIB). The plastid DNAs are released from organelles and eliminated with an osmotic buffer by washing and centrifugation. The purified nuclei are then lysed and further cleaned by organic extraction, and the genomic DNA is precipitated with a high concentration of CTAB. The highly pure, high molecular weight gDNA is extracted from the nuclei, dissolved in a high pH buffer, allowing for stable long-term storage.

**Conclusions:**

This method is unique and avoids the use of embedding in agarose, which dramatically reduces time (4–8 h versus days), complexity, and materials cost. This procedure can be used on essentially any plant species and tissue stage. Here we describe a case study and a simple method to rapidly prepare high molecular weight gDNA from Upland cotton, blackgrass, and strawberry suitable for single-molecule sequencing.

## Background

Technological advancements have fueled a rapid shift in genome mapping and sequencing strategies. Second generation DNA sequencing technologies (short read), such as Illumina and Ion Torrent are beginning to become shadowed by long read length capabilities and improving accuracies of 3rd generation technologies, such as 10 × Genomics [1], Pacific Biosciences [2], and Oxford nanopore [3]. These long read platforms have facilitated a shift in whole genome sequencing strategies from hybrid approaches (short + long-read) to a singular platform for de novo data collection and/or resequencing. Genomic physical mapping has also seen a transition from hierarchical BAC-by-BAC approaches to optical mapping, with the primary platform being the Saphyr^®^ [4]. As these single-molecule technologies continue to evolve, the need for high purity, extremely large, in-tact genomic DNA also continues to rise. Most plant species are rich in polyphenolic compounds, polysaccharides, tannins, and other secondary metabolites that make quality DNA extraction difficult or even impossible. Typically, for high-molecular-weight (HMW) genomic DNA (gDNA) extractions, selected plant materials should be young, expanding leaf tissue without pathogen and/or pest infection. The most common extraction procedures follow these basic rules: (1) Cell wall disruption to release nuclei and the organelles. This step is commonly performed by grinding tissues in the presence of liquid nitrogen; (2) Breaking the organelles to release organellar DNA, other than the nuclei, with an osmotic nuclear isolation buffer (NIB) containing 0.5% Triton X-100; (3) A wash with the NIB to release the total DNA from all organelles; (4) Lysis of the nuclear membranes with a detergent to release the target gDNA, which should be protected from nucleases and physical shearing. These steps are not trivial and often times the resulting gDNA is highly oxidized, sheared, or co-precipitated with polysaccharides or other compounds that interfere with yield or downstream enzymatic reactions.

Nuclei isolation from plant leaves has been widely applied to many plant species [5, 6]. However, this method is time consuming, costly, and is often difficult to retrieve sufficient yield and purity of resulting DNA. These procedures often require high concentrations of antioxidants to reduce or partially inhibit the oxidation of the gDNA; while high salt concentrations (NaCl) are typically used to disassociate the bond between polysaccharides and carbohydrates, with usually unsatisfactory results. These techniques do not consistently work for many plant species that are rich in these troublesome compounds. The cetrimonium bromide (CTAB) precipitation method is much more efficient in eliminating polysaccharides from the plant tissues [7], but is not typically used in the isolation of megabase-sized DNA because of physical shearing during precipitation and the contamination of the target nuclear DNA by plastid DNA. Aside from oxidation and co-precipitation/association with unwanted compounds, another primary issue in HMW DNA extraction is the physical shearing that results from grinding, exposure time to air and warm temperature before the ground tissue is completely mixed with NIB buffer, DNA precipitation procedures, dissolving and storage. Conventional protocols minimize mechanical breakage of gDNA by embedding the nuclei in an agarose matrix (plugs) and conducting protein digestion and other enzymatic activities in the plugs [6]. However, single-molecule sequencing devices require an aqueous solution of gDNA that is highly concentrated in a small volume. Genomic DNA extraction from the plugs is conducted by either electroelution or enzymatic digestion of the agarose; where electroelution typically results in very dilute gDNA solutions with low total mass concentrations and large volumes, while the enzymatic techniques often lead to co-precipitation of agarose.

Here, we present a simple, nuclei-CTAB method for the extraction of large, in-tact HMW gDNA from plants. This procedure is simple, economical, rapid, and offers gDNA that is ultra-pure from oxidative and polysaccharide compounds and contaminating plastid DNAs. This procedure has been successfully used on Upland cotton, *Gossypium hirsutum,* (a species rich in polyphenolics), blackgrass, *Alopecurus myosuroides,* (a grass species with high amounts of carbohydrates), and strawberry, *Fragaria* x *ananassa,* (a Rosaceous species rich in polyphenolics, polysaccharides, flavonoids, and secondary metabolites); we also provide suggestions for overcoming the intractable problems frequently encountered in megabase-sized DNA preparation. We conclude that the nuclei-CTAB method is a choice for the preparation of HMW gDNA from various plant species for many research goals, including the construction of large-insert BAC and BIBAC libraries, genome mapping, single-molecule sequencing and long-range genome analysis.

## Results

### Yield, quantity, and quality of the isolated gDNA

**Fig. 1:**
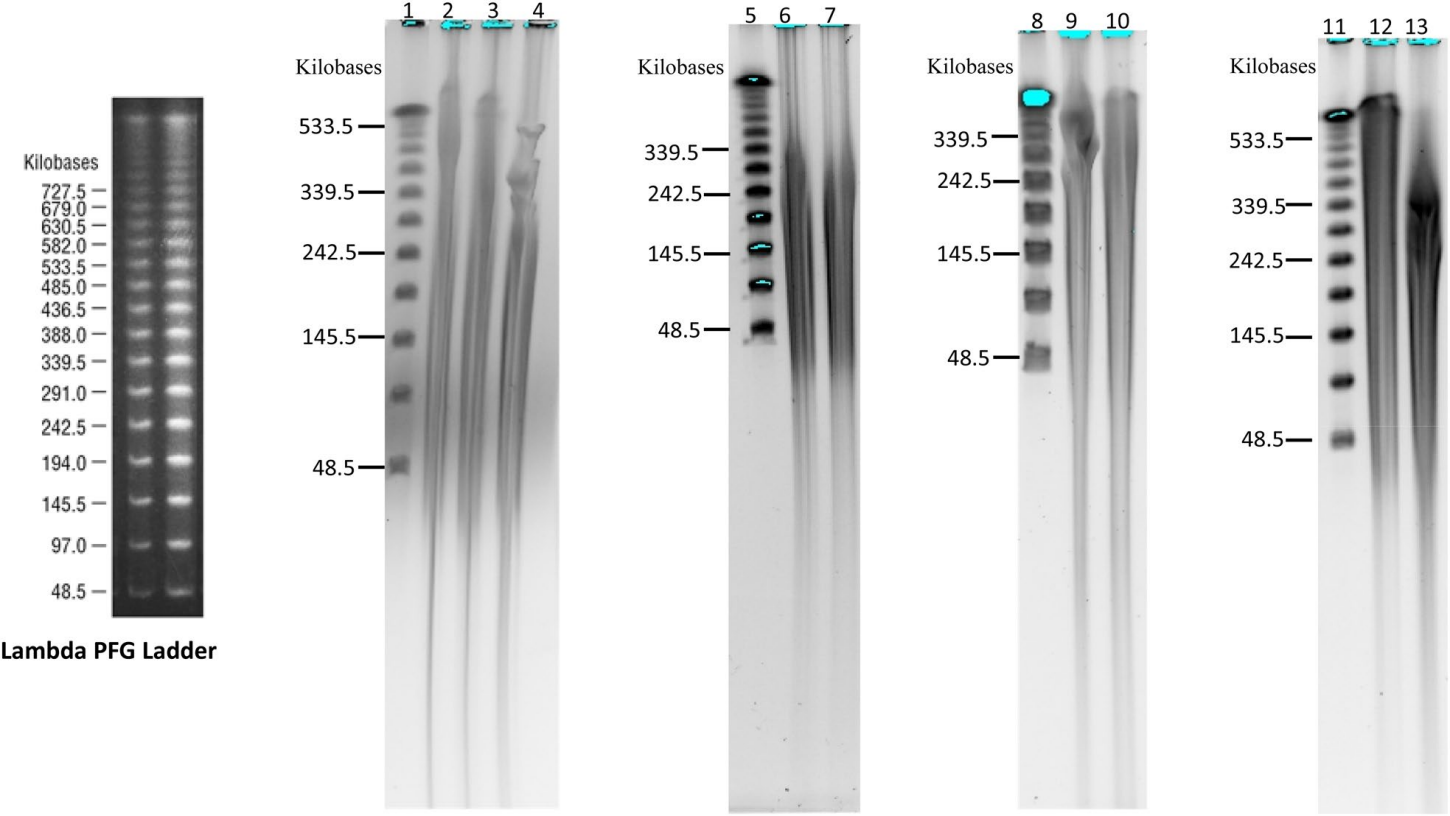
The high-molecular-weight genomic DNA fractionation by Pulsed-field gel system (CHEF system; initial SW time: 1.0, final SW time: 40.0; voltage, 6 V/cm; included angle: 120; running at 14 °C for 16–24 h in 0.5 × TBE buffer). Lane 1, 5, 8 and 11 Lamda PFG Marker; lane 2, cotton CSX8303; lane 3, blackgrass grass; Lane 4, cotton UGA230; lane 6 and 7, strawberry genotype Royal Royce; lane 9, cotton Coker, lane 10, cotton 94-L25 genomic DNA; lane 12, High concentration (900 ng/µl) cotton Jin668 gDNA stored at − 20 °C for 16 month, lane 13, Low concentration (200 ng/µl) cotton Jin668 gDNA stored at − 20 °C for 16 month

With this protocol, we have successfully prepared high-quality megabase-sized DNA from cotton (species: *Gossupium hirsutum* cv’s: TM1, UGA230, CSX8303 and UA48), blackgrass (*Alopercurus mysuroides*), and strawberry (*Fragaria ananassa*). For data presented in this work, DNA was prepared from young leaves for cotton and blackgrass, mixed meristematic tissue and leaves for strawberry. The gDNA yield from each of these species is greater than 100 micrograms of gDNA per 10 grams fresh tissue, which meets the needs for single-molecule technologies. DNA was quantified by measuring the concentration using a NanoDrop 8000 Spectrophotometer (Thermo Fisher Scientific, USA) and Qubit^®^ 2.0 Fluorometer with Qubit dsDNA BR Reagent (Thermo Fisher Scientific/Invitrogen, USA). The range of absorbance ratios of A260:280 is 1.80–1.84, and of A260:230 is 1.94–2.0. The DNA quantity measured by NanoDrop 8000 Spectrophotometer and Qubit^®^ 2.0 Fluorometer is similar, and in each sample is greater than 500 ng/ul. The high molecular weight DNA size distribution was examined by pulsed field gel electrophoresis on 1.0% agarose gels stained with ethidium bromide. The majority of the gDNA fragments are distributed between 100 and 1000 kb (Fig. 1), and digestible by restriction enzyme.

## Method

1. Selection and preparation of source tissue: Young expanding leaf and/or shoots material is optimal and should be dark treated for 12 ~ 24 h prior to harvesting to reduce photosynthetic byproducts.
2. Grind ~ 10 g of the frozen or fresh tissue (Tissue weight: NIB buffer ≤ 1:10) into fine powder in the presence of liquid nitrogen with a mortar and pestle, and then transfer the fine powder into an ice-cold 500-ml flask containing ~ 120 ml of the NIB buffer (with 0.5% final concentration of 2-Mercaptoethanol, BME), immediately mix well.
3. Gently mix the contents of the flask on ice for 10 min at 100 × rpm.
4. Filter mixture through two layers of cheesecloth and two layers of Miracloth sup-ported by 15-cm diameter-funnel. Collect the remaining nuclei suspension by squeezing the cheesecloth-wrapped pellet gently with gloved hands.
5. Aliquot the filtrates into two 50-ml conical tubes, pellet the homogenate at 2400×*g*, 4 °C for 12 min, discard the supernatant.
6. Resuspend the pellet using a small paint brush with 20 ml of ice-cold NIB for each tube, combine into one tube; pellet the nuclei at 2400×*g*, 4 °C for 6 min.
7. Repeat the step 5 for 1–3 times until the green color is removed and the suspension is clear.
8. Discard the supernatant; add 0.5–2 ml of ice-cold NIB buffer to each conical tube, resuspend the pellet completely gently using a small paint brush.
9. Pour 20 ml of 2 × CTAB (65 °C) buffer containing 0.5% BME and immediately mix well, incubate at 65 °C for 10 min, then cool down to RT.
10. Extract with equal volume of chloroform by gentle shaking (40 ~ 60 rpm) or by gentle inversion, spin at 2400×*g* for 10–30 min at room temperature.
11. Transfer 20 ml of the supernatant to a new tube, add 2 ml of 10% CTAB buffer (pre-warmed at 65 °C), mix well gently, incubate at 65 °C for 3–5 min. Cool down to RT.
12. Extract with same volume of chloroform by gently shaking (40–60 rpm) or by gentle inversion. Spin at 2400×*g* for 10–30 min at room temperature.
13. Transfer 15 ml of the supernatant into a new conical tube.
14. Add the same volume of 1 × CTAB precipitation buffer (in this case, add 15 ml of 1 × CTAB precipitation buffer), mix very gently by inverting to precipitate the genomic DNA.
15. Centrifuge at 2400×*g* for 5 ~ 30 s, discard the supernatant and resuspend the pellet using 600 µl of TE high-salt solution.
16. Transfer the suspension in a 2-ml tube, add 1.2 ml of ethanol, mix gently till DNA precipitated, and leave the tube at room temperature for 5 min.
17. Centrifuge at 3000×*g* for 5 min.
18. Wash 2 times with 70% ethanol.
19. Remove the residual 70% ethanol, air dry DNA for 5 min and re-suspend in 200–400 µl of 0.1 × TE with RNaseA.

## Discussion and key notes

1. Dark treatment is crucial for reducing carbohydrate, polysaccharide, and polyphenolic compound production. For species that typically produce high amounts of these compounds, e.g., strawberry, 48 h is recommended. For high quality gDNA, tissue should be harvested and stored at − 80 °C. Storage times longer than 1 year, may result in degraded gDNA. For some species with low nuclei yield, or with low contents of the polyphenolic compounds, polysaccharides, tannins, and other secondary metabolites, for example, *Arabidopsis*, an increased amount of tissue is recommended, e.g., 20–40 g should be used in this protocol. For fiber-rich species, for example, maize, grinding the tissue to a fine powder is key.
2. The tissue must be mixed well in the flask very quickly; if the frozen tissue clumps, break it apart with a spatula or glass rod.
3. Miracloth should be outside (attached to the funnel) and cheesecloth be inside. Filtration step should not take more than 5 min.
4. The supernatant (or waste solution) containing BME must be stored as hazard waste in a fume hood.
5. Resuspend the pellets very carefully with a paint brush. The paint brush can be sterilized with 70% ETOH (make sure it is dry with no residual ethanol) or by UV-nicking.
6. The resuspension solution showing green color, suggests the chloroplasts are not completely disrupted by NIB. In this case, continue to repeat the wash, pellet and resuspend several times to remove the unwanted plastid DNA.
7. The final volume of the nuclei resuspension should not more than 2-ml.
8. 20 ml of 2 × CTAB must be poured into the nuclei suspension very quickly, and immediately mix well by vortexing for 1–2 s. During incubation, invert the tube several times gently.
9. The chloroform extraction should be executed quickly within ~ 5 min.
10. When pipetting nuclei solution from organic extraction, using cut (large orifice) 5-ml tip.
11. Volume ratio of the 10% CTAB versus the supernatant should be 1:10.
12. During the last organic extraction, take caution not to disturb the interphase. Do not try to transfer the entire aqueous phase. If the interphase was transferred, spin @RT for another 10 min, then transfer the aqueous phase into a new tube).
13. When adding the 1 × CTAB precipitation buffer, and after mixing around 10 times by slow inversion, you should see the silk-like gDNA precipitate. When this becomes evident, do not continue to invert. This will aggregate the gDNA which will become too dense to dissolve.
14. If the pellet is difficult to dissolve, incubate at 56 °C for 5–10 min, and then incubate at 4 °C overnight in a refrigerator.
15. If there is a visible undissolved slurry before adding ethanol, spin at 3000×*g* for 5 min, then transfer the liquid phase into a new 2-ml tube for DNA precipitation.
16. Do not let the pellet become too dry. If the pellet is difficult to rehydrate, incubate at 4 °C overnight.

### Reagents

- 2-Mercaptoethanol (BME, Fisher Scientific)
- Ethidium bromide (Fisher Scientific)
- *Hin*dIII (NEB)
- Midrange Lambda ladder PFG marker (NEB)
- Lambda ladder PFG marker (NEB)
- Yeast chromosome PFG marker (NEB)
- Na_2_ EDTA (VWR)
- NaCl (VWR)
- NaOH (VWR)
- Liquid nitrogen,
- Spermidine trihydrochloride (Sigma-Aldrich)
- Spermine tetrahydrochloride (Sigma-Aldrich)
- Sucrose, molecular biology grade (VWR)
- Triton X-100 (Fisher Scientific)
- Trizma base (Fisher Scientific)
- Cyltrimethylammonium bromide, CTAB (MP Biomedicals)
- Polyvinylpyrrolidone, PVP (MV: 40,000) (Sigma-Aldrich)

### Equipment and supplies

- Mortar and pestle, 0.5–1 L in volume (CoorsTek)
- 50 ml Conical tube (VWR)
- Cheesecloth (VWR), cut into 25 × 25 cm^2^ pieces
- Miracloth (Calbiochem, CAT# 475,855), cut Miracloth into 25 × 25 cm^2^ pieces
- Funnel, 15 cm in diameter
- Glass flask, 500 and 1000 ml
- Magnetic stir bar
- Gloves (VWR)
- Paint brushes (12 Kid Art Brushes, Pinceaux d’artiste), sterilize by UV
- Pipette tips, 200 μl, 1000 μl, 5000 μl
- Swinging bucket centrifuge (Allegra X-15R, Beckman)
- Water bath (Precision)
- Pulsed-field gel electrophoresis (PFGE) systems: the CHEF-DR III System, Cat # 170-3695, 170-3700, and other parts (from 170-3690 to 170-3703, Bio-Rad)

### Buffer setup

**HB (homogenization buffer) stock (10×)**

For 10 × stock (100 mM Trizma base, 800 mM KCl, 100 mM EDTA),

Add

12.1 g of Trizma base,

59.6 g of KCl,

37.2 g of Na2 EDTA,

to ~ 800 ml of ddH2O in a 1-l PYREX bottle. Stir until dissolved. Adjust the pH of the solution to 9.2 with NaOH and bring to a final volume of 1000 ml in a 1000-ml graduated cylinder. Transfer the solution to a glass stock bottle and store it at 4 °C. The 10 × HB stock may be stored at 4 °C for up to 1 year.

**HB solution (1×)**

To prepare stock solution (1 × HB, 0.5 M sucrose, 1 mM spermidine trihydrochloride, 1 mM spermine tetrahydrochloride)

Add

100 ml of 10 × HB stock

171.2 g of sucrose to ~ 700 ml of ddH2O

0.255 g of spermidine trihydrochloride

0.348 g of spermine tetrahydrochloride

in a 1-l PYREX bottle. Stir until dissolved. Bring to a final volume of 1000 ml in a 1000-ml graduated cylinder. Transfer the solution to a glass stock bottle and store it at 4 °C for up to 3 months.

**Triton X-100 (20% (vol/vol) Buffer**

For 20% (vol/vol) Triton X-100 buffer [1 × HB, 0.5 M sucrose, 20% (vol/vol) Triton X-100],

Add

20 ml of Triton X-100

10 ml of 10 × HB stock

17.15 g of sucrose to ~ 60 ml of ddH2O in a 100-ml beaker.

Stir until dissolved. Bring to a final volume of 100 ml in a 100-ml graduated cylinder.

Transfer the solution to a glass stock bottle and store it at 4 °C. The 20% Triton X-100 stock may be stored at 4 °C for 1 year or longer without major problems.

**NIB buffer** (Nuclei Isolation Buffer)

Make the buffer (1 × HB solution, 0.5% Triton X-100, 0.5% (vol/vol) 2-Mercaptoethanol) just before use. The volume of the buffer needed is 10–15 ml per gram weight of the sample, including nuclei isolation and sub-sequent washes.

For 200 ml of the buffer,

Mix

195 ml of 1 × HB

5 ml of 20% (vol/vol) Triton X-100 buffer

Mix well and keep it at 4 °C or on ice.

Immediately before use, add 1 ml of BME to the buffer.

### Critical

Always use freshly made buffer for megabase-sized DNA isolation

**2 × CTAB buffer (pH = 9.2)**

2% CTAB (w/v)

100 mM Tris

20 mM EDTA (ethylenediaminetetraacetic acid)

1.4 M NaCl

1% PVP (polyvinylpyrrolidone) M_r_ 40000

**10% × CTAB buffer**

10% CTAB

0.7 M NaCl

**1 × CTAB precipitation buffer (pH = 9.2)**

1% CTAB

50 mM Tris

10 mM EDTA

**High salt TE buffer (pH = 9.0)**

10 mM Tris

1 mM EDTA

1 M NaCl

**0.1 × TE buffer (pH = 9.0)**

1.0 mM Tris

0.1 mM EDTA

## Conclusion and discussion

Here we present a simplified method for the extraction of large, in-tact, high-quality genomic DNA for plant species. This method avoids the need for embedding nuclei in agarose and offers a simplified purification procedure that is aimed to dissociate the gDNA from co-precipitating compounds, such as polysaccharides, carbohydrates, polyphenolic compounds, and other problematic secondary metabolites. The method does not require specialized equipment, and can be completed in a day, versus multiple days to a week. The resulting genomic DNA is of suitable quality for single-molecule sequencing and mapping technologies.

## Data Availability

Data sharing is not applicable to this article as no datasets were generated or analyzed during the current study.
